# RETurn of the Gene. Post scriptum

**DOI:** 10.1530/ERC-26-0178

**Published:** 2026-08-31

**Authors:** Tom R Kurzawinski, Kate Newbold, Neil Q McDonald

**Affiliations:** ^1^ Consultant Endocrine Surgeon, University College Hospital and Great Ormond Street Hospital for Children NHS Trusts, Honorary Assistant Professor University College London, London, United Kingdom; ^2^ Thyroid Unit, The Royal Marsden NHS Foundation Trust, London, United Kingdom; ^3^ Signalling and Structural Biology Laboratory, Francis Crick Institute, London, United Kingdom; ^4^ Institute of Structural and Molecular Biology, School of Natural Sciences, Birkbeck College, London, United Kingdom

**Keywords:** RET, MEN2, medullary thyroid cancer, primary hyperparathyroidism, pheochromocytoma

The idea of the RET@Thirty: Three Decades of Remarkable Progress special collection, now completed in *Endocrine-Related Cancer*, developed in two meetings that took place in the United Kingdom and were led by the UK RET Clinical and Research Collaborative Network. First of them, ‘**RET@Crick**’ (https://www.crick.ac.uk/whats-on/ret-crick), took place in July 2019 at the Crick Institute in London, and the second, ‘**RET@Thirty: Three decades of remarkable progress**’ (https://www.endocrine-abstracts.org/ea/0094/abstracts/ret-30-symposiumsessions/section/), took place in November 2023 at the Annual Congress of the Society for Endocrinology in Glasgow. Both meetings aimed to facilitate the exchange of ideas and collaborative initiatives leading to innovative projects and grant applications by bringing together scientists and clinicians. When *Endocrine-Related Cancer* offered us the opportunity to put together a selection of articles from these two meetings, the Editors set out enthusiastically to fulfill the task of inviting contributions from RET experts from around the world. However, we soon realized that, unknowingly and unintentionally, our choice of the title **RET@Thirty** (which was chosen in 2023) might suggest that the *RET* gene was discovered in 1993. This, however, is not the case; *RET* was discovered in 1985, and our title intended to refer to the moment in time when *RET* was associated with the multiple endocrine neoplasia type 2 (MEN2) syndrome (1, 2, 3). This pivotal moment in the RET story is wonderfully described in Bruce Ponder and Tom Kurzawinski’s opening editorial, ‘**RET@Thirty: the story behind identification of *RET* mutation as the cause of MEN2 and what it meant to clinical practice**’ (4).

This has led us to an interesting question: how old is the *RET* gene after all? *RET* is an important gene that plays a significant role in developmental and evolutionary biology. It is highly conserved throughout the evolution of vertebrates (from fish to mammals), indicating its critical biological function. *RET* is an innovation of vertebrates, as before their evolution in the Cambrian period around 500 million years ago (Mya), only tyrosine kinase-like genes, known as ancestral receptor tyrosine kinases (RTKs), existed in metazoans (5).

Those ancient RTKs (600–900 Mya) had extracellular ligand-binding domains, transmembrane regions and intracellular TK domains. During evolution, vertebrates ‘built’ *RET* as we know it, with the acquisition of cadherin-like domains (CLDs) and a cysteine-rich domain (CRD). A pair of CLDs in RET mediate extracellular binding to a co-receptor (GFRα) and, together with the CRD, facilitate receptor stabilization and ligand-binding interactions. RET was built during evolution through gene duplication, and the domain structure was assembled through exon shuffling and modular acquisition/recombination. RET signaling co-evolved with other molecular components as a ‘package’ with the co-receptor GFR and glial cell line-derived neurotrophic factor (GDNF) family ligands (5). The arrival of RET enabled increased anatomical and physiological complexity in vertebrates; its signaling is crucial in neural crest development (a fundamental innovation of vertebrate evolution), kidney organogenesis, formation of the enteric nervous system, maturation of spermatogonial stem cells and expansion of hematopoietic stem cells. *RET*, as we know it, is therefore about 500 million years old and deserves to be known as a Senior Proto-oncogene! A more appropriate moniker may be ‘Chameleon Gene’, reflecting how its allelic variants cause distinct diseases, with gain-of-function mutations making it a dominant oncogene, while loss-of-function recessive mutations being causal of developmental disorders. The *RET* gene encodes a tyrosine kinase receptor, which, through the GDNF ligands, activates several downstream pathways, such as MAPK/ERK, PI3K/AKT and PLC, and regulates cell proliferation, differentiation, migration and survival. With such activities under its control, it is not surprising that germline and somatic *RET* gene mutations and fusions are responsible for a range of diseases, including MEN2, sporadic medullary thyroid cancer, pheochromocytomas, lung cancers, Hirschsprung’s disease, intellectual disabilities, congenital kidney and urinary tract abnormalities. Although individually rare, collectively they carry a significant burden to the patients and healthcare systems because of their complexity and multiple comorbidities.

The RET@Thirty special collection aims to sum up current knowledge about RET and to celebrate the remarkable progress in this field since the discovery of the gene–disease association. It includes two editorials and twelve mini reviews addressing the multifaceted investigations of RET and their transforming impact on patients; molecular and biological insights from basic research; and clinical practice in endocrinology, surgery, oncology and other medical specialties.

‘**Living with a *RET* gene mutation**’ is written by patients diagnosed with MEN2 and their caregivers who are frequently parents (6). The editors would like you to read this article first, and if you do not have time to read other articles in the whole special collection, you must read this one. No one can tell the story of what it is like to live with MEN2 as well as those who have skin in the game. They understand best what obstacles must be overcome to get the correct diagnosis, the post-diagnosis impact, the frustrations of fluctuating calcitonin levels and the never-ending concerns of MTC recurrence or development of pheochromocytomas. Patients with MEN2b have other worries, such as further bowel surgery, changes in facial features and musculoskeletal pain. The authors tell the stories of certain mutations not always following the rules of genotype/phenotype correlations (e.g. at codon p.V804M), being told bad news in an inappropriate setting, overtreatment and the impact on family relationships following diagnosis. Patients with MEN2 and the organizations that represent them have called for centralization of the care for patients with this condition, as they know first-hand about the difference of being looked after by, as they refer to them, ‘*centres of expertise and experience*’.

Genomic testing has expanded greatly in the past decade, moving on from limited access within clinical genetics services into mainstream care, *RET* mutations being no exception. Germline and/or somatic *RET* variants are routinely tested early in a patient’s disease-related journey, to determine whether there is a heritable cause and to facilitate mutation-specific clinical care. ‘**Genomic testing for *RET* in the clinic: UK and global perspective**’ (7) is an authoritative and informative mini review about the current state of genomic *RET* testing and explores issues of great significance in this constantly shifting field. It discusses current germline and somatic testing strategies in individuals with mutant *RET*-associated disease and addresses issues such as who to test, which test to choose, genomic analysis, reporting, test outcomes (including variants of uncertain significance or incidental findings), how to follow-up on the test results (care of individuals and family), reproductive options and the still controversial new initiatives of population screening, such as newborn genomic testing programs. It considers challenges of delivering rapid, reliable and equitable access to genomic testing and gives many examples of online resources, which can be accessed in the UK through a national genomic service, which now provides the entire English population with access to genetic testing for inherited rare diseases and cancer (similar services exist in Scotland, Wales and Northern Ireland).

Within a few years of the *RET* discovery, strong genotype/phenotype correlations in patients with MEN2 was noticed and this phenomenon has since remained central to clinical practice. The review ‘**Genotype/phenotype correlations in multiple endocrine neoplasia type 2**’ (8) discusses the main correlations of the numerous pathogenic variants of *RET* identified so far and the potential factors that might influence these correlations. Such a discussion is perhaps most relevant for MTC, and this mini review highlights unexpected situations when patients with high-risk variants present with non-aggressive MTC and patients with moderate-risk variants present with very aggressive disease, suggesting that factors other than the main *RET* variant may modify the natural history and genotype/phenotype correlations. Simple paradigms of MTC aggressiveness and prognosis linked to specific *RET* mutations have also been challenged, especially in cases with high- and moderate-risk mutations, where rates of remission, metastatic disease and death are similar in both groups when age at diagnosis of MTC rather than chronological age is considered. It seems that the concepts of ‘risk’, ‘aggressiveness’, ‘onset’ and their relation to specific *RET* mutations are slowly undergoing an evolution in their interpretation. We also need to know which pathophysiological mechanisms driven by *RET* mutations are responsible for modifying the natural history of the disease, including possible factors such as single nucleotide polymorphisms of *RET*, emergence of somatic mutations, vertical transmission, consanguinity and the environment. All these aspects of RET are discussed in this article (8).

Pathology from *RET* mutations encompasses a vast array of non-endocrine organs (striatal dopaminergic, urinary and enteric nervous systems) and endocrine glands not affected in MEN2, such as the pituitary gland (somatotrophinomas). RET is expressed in somatotrophs and pituitary stem cells and acts as a dependence receptor, where its ligand GDNF promotes survival and cell proliferation at the expense of controlling differentiation through the PIT1 transcription factor. PIT1 regulates cell differentiation and normal GH secretion, and insufficient GDNF levels trigger apoptosis through complex intracellular RET processing stimulating PIT1 expression. ‘**RET signalling in the pituitary: a double-edged sword for differentiation, apoptosis and therapeutic strategies in acromegaly**’ (9) discusses the role of RET in the pituitary gland, focusing on its regulation of two opposing pathways – survival and apoptosis – and explores the potential therapeutic implications of these pathways for acromegaly patients.

Charting the remarkable progress in our understanding of the molecular biology of RET constitutes a large and exciting part of this collection. Molecular aspects of RET are discussed in four separate contributions, covering its structure, biology, signaling and function with the aim of highlighting the current state of scientific knowledge and potential roles of RET in human biology.

Demonstrating that function follows form, the structure of RET reveals clues to its evolutionary past and how its architecture encodes a recognition module for binding to the GFR co-receptor and GDNF family ligands (GFLs). The review ‘**RET receptor tyrosine kinase architecture, assemblies, and activation**’ (10) describes the latest findings about the modular organization of the RET receptor emphasizing the unusual arrangement of cadherin-like domains, which have diverged from canonical cadherin domains (with some losing their calcium-binding sites). The intrinsic plasticity and shape complementarity of RET and its ligands allow capture of each pair of GFL–GFR heterodimers with a precise spatial separation of RET dimers driving activation. Evidence for dimeric and higher-order assemblies suggests that emergent properties and feedback loops remain to be uncovered. The current evidence is described for how the RET receptor is activated on binding to the GDNF family ligand and GFR co-receptor, resulting in signal transduction upon persistent ligand interaction. Future progress may come from looking at intact RET protein complexes *in situ* within living cells, capturing and visualizing distinct functional states of RET, and assessing the kinetics of RET-driven signaling cascades leading to integrated cellular decision making.

Understanding the full repertoire of RET biology requires a knowledge of the complete set of its ligands and careful analysis of phenotypes arising from genetically manipulating the levels of RET signaling components. The review ‘**Biology of RET receptor and its ligands: focus on the nervous system**’ (11) describes recent progress uncovering growth differentiation factor 15 (GDF15) as an additional ligand of RET that has revealed new biological roles in metabolism and stress signaling, thus broadening the scope of RET biology. This brings with it new opportunities for therapeutic intervention in RET signaling. RET is well established as a target for inhibition by small molecules in cancer contexts as covered by other reviews in this *Endocrine-Related Cancer* special collection. However, Ret also presents as a target for modulation by activation in neurodegenerative contexts, such as Parkinson’s disease, through binding to GFLs. Here, therapeutic delivery remains a problem as described in this review. Efforts to identify small molecules with better delivery and diffusion properties, including GFL mimetics, to drive RET activation may ultimately prove to be more successful.

RET operates as a major signaling hub maintaining homeostasis in several metazoan tissues. As fundamentally allosteric proteins, receptor tyrosine kinases, such as RET, must respond robustly and accurately to ligand occupancy outside the cell, leading to receptor activation within the cell. Central to RET activation are specific tyrosine phosphorylation sites that act as handles upon which to assemble specific signaling cascades. The review ‘**Mechanisms of RET-mediated signal transduction**’ (12) addresses current knowledge on specific signaling cascades and their associated phosphotyrosine sites. Since what goes up must come down, these same phosphotyrosine handles can also act as negative regulators promoting either dephosphorylation or ubiquitination, leading to RET degradation. The same review describes known mechanisms and key factors involved in the negative regulation of RET signaling (12).

Unlike most familial cancers, which are caused by tumor suppressor genes, RET is a proto-oncogene, and a single mutant allele can drive oncogenesis. The review ‘**Cellular mechanisms of RET receptor dysfunction in multiple endocrine neoplasia 2**’ (13) describes with incredible clarity mechanisms of MEN2A and MEN2B pathobiology and how distinct patterns of RET amino acid substitution mutations affect tumor prevalence, onset, aggressiveness and the associated constellation of other disease features. It draws to our attention that *RET* gain-of-function point mutations not only cause intrinsic biochemical changes in the RET structure and activation but also trigger extrinsic effects that alter RET cellular location, leading to its prolonged or mislocated activity. Specific *RET* mutations alter molecular and cellular function differently and ‘fine-tune’ these effects conferring unique patterns of disease phenotype. For example, substitutions of more distal cysteines (e.g. codons C609, C618 and C620) have pronounced effects on RET protein folding, which impairs maturation to the plasma membrane localization and subsequent kinase activity that may lead to later disease onset or reduced penetrance. Also discussed in this review are other modifying factors, such as increased expression of the wild-type (WT) RET receptors in both MEN2 subtypes, which might slow disease course, and how MEN2-*RET* mutant tumors could influence their microenvironment by affecting host immune–inflammatory responses (13).

Diagnostic and therapeutic implications of genotype/phenotype correlations in patients with the MEN2 syndrome and its impact on the timing and extent of thyroid, adrenal and parathyroid surgeries are reviewed in ‘**MEN2: surgical precision in the era of precision medicine**’ (14). It is possibly the longest ‘mini review’ in the history of scientific publishing, its length perhaps justified by the fact that surgical resections remain the most effective method of preventing the development of tumors or curing them in three distinctive endocrine organs. It reviews recent scientific discoveries and technological advances that made the surgical decision process more precise. It discusses how genetic analysis allows us to predict the onset and aggressiveness of mutant *RET*-related pathologies with a fair degree of accuracy and how biochemical monitoring provides regular updates on the transformation of endocrine cells in target endocrine organs, and together with imaging, helps to decide on the timing and extent of surgery. Its main argument is that calibrating the extent and type of surgery able to cure but do minimal harm, timing and performing it well is the art of surgical precision in MEN2 patients. This leads to safer and less invasive interventions, which result in fewer complications, less trauma and better functional outcomes.

Novel cancer therapies targeting Ret alterations are addressed by two mini reviews alongside a special article on the mechanisms of resistance to these systemic therapies: ‘**Targeting *RET* in medullary thyroid cancer**’ (15) and ‘***RET* fusions in solid cancers**’ (16). The two mini reviews discuss hugely significant and positive progress in the management of these cancers in the advanced stage. RET-targeted therapies with selpercatinib and pralsetinib have shown high response rates and are well tolerated with low-grade toxicities in the majority. This has resulted in a notable improvement in the quality of life of patients with reduced disease-related symptoms and facilitated improvements in progression-free survival. Rates of toxicities are lower and less severe in patients on RET-directed therapy compared with previous standards of care. The disease will, however, inevitably become resistant to the first-line RET-directed therapy and therefore, it is critical that the mechanisms of emergent resistance are explored and defined alongside development of novel agents or combination therapies to mitigate the disease. As described in ‘**Mechanisms of resistance to RET-directed therapies**’ (17), this is an area of active research and progress that is key to ongoing improvement in outcomes for patients with *RET*-altered cancers.

This *Endocrine-Related Cancer* special collection is a required ‘close reading’ of the *RET* proto-oncogene. It celebrates the scientific endeavors over the past 30 years, which have explored the fundamental biology and function of RET, the consequences of pathological alterations of *RET* and the development of clinically game-changing therapeutic options directed at mutant *RET*-driven cancers. It also explores potential new advances in this field that are anticipated in the future and, hopefully, will inspire a new generation of scientists and clinicians in their quest for a better understanding of RET molecular mechanisms, better clinical therapy pathways and improved patient outcomes.

## Declaration of interest

The authors declare that there is no conflict of interest that could be perceived as prejudicing the impartiality of the work reported.

## Funding

This work did not receive any specific grant from any funding agency in the public, commercial or not-for-profit sector.
